# Comparison of Intramedullary and Extramedullary Fixation Results in Subtrochanteric Femur Fractures

**DOI:** 10.7759/cureus.49258

**Published:** 2023-11-22

**Authors:** Ersin Şensöz, Gultekin Cecen

**Affiliations:** 1 Orthopedics, Dr. Lutfi Kirdar Kartal City Hospital, Istanbul, TUR; 2 Orthopedics and Traumatology, Bahçeşehir University Medical School, Istanbul, TUR; 3 Orthopedics and Traumatology, VM Medical Park Pendik Hospital, Istanbul, TUR

**Keywords:** extramedullary fixation, intramedullary fixation, subtrochanteric fracture, retrospective, proximal femur fractures

## Abstract

Objective: The subtrochanteric femur is a load-bearing region where deforming forces are effective. These fractures are difficult to treat for orthopedic surgeons. In this article, we aimed to compare intramedullary (IM) and extramedullary (EM) fixation methods for these fractures that do not have gold standard treatment.

Methods: Eighty-five patients were retrospectively evaluated. Patients with IM implants (IM femoral nail, proximal femoral nail) were grouped as Group 1, and patients with EM implants were grouped as Group 2 (95° condylar plate, locked anatomical proximal femoral plate). Patients' age, gender, fractured hip side, etiology, anesthesia type, preoperative waiting time, follow-up period, fracture type, Harris hip score (HHS), and mechanical complications were examined.

Results: The mean age of the patients was 44.13 years. There was no difference between the groups in terms of age and gender. The mean follow-up period of the patients was 13.28 months. Falling from a height was the most common cause of subtrochanteric fracture. IM fixation was applied to 62 patients, and EM fixation was applied to 23 patients. No significant difference was found between Groups 1 and 2 in non-union, delayed union, implant failure, shortness rates, and HHS.

Conclusion: Both IM and EM fixation methods have advantages and disadvantages in treating subtrochanteric fractures. These methods yield similar results in both groups in our study. The issue of fixation methods remains controversial in the literature, and further studies are needed on this subject.

## Introduction

Fractures within 5 cm of the lesser trochanter in the proximal femur are called subtrochanteric fractures. Subtrochanteric fractures constitute 25% of all hip fractures and 7-4% of all femur fractures [1-5]. They occur with injuries in young, high-energy patients and traumas in low-energy older individuals. Since subtrochanteric fractures have been defined, many fractures are difficult to treat and have a high complication rate. Deforming muscle forces in the proximal femur make fracture reduction difficult during surgery and may lead to impaired union or non-union after surgery.

In the past, open reduction and plate fixation were common treatments for subtrochanteric fractures. Over time, intramedullary (IM) nails have been applied, and their advantages, such as biomechanical superiority, minimal invasiveness, and shortening of operation time, have been noticed [2,4]. While IM fixation has been a common treatment for femoral diaphyseal fractures, the approach to subtrochanteric fractures remains unclear.

There are few studies comparing IM and extramedullary (EM) fixation methods in subtrochanteric fractures. In the literature, treatment of these fractures has involved different fixation methods, but there is still no consensus. Therefore, the aim of this study was to evaluate the results of treatment of subtrochanteric femoral fracture using IM (IM femoral nail, proximal femoral nail (PFN)) and EM (95° condylar plate, locked anatomical proximal femoral plate) fixation methods.

## Materials and methods

All procedures involving human participants in this study were performed in accordance with the ethical standards of the institutional and national research committees and with the 1964 Helsinki Declaration and its later amendments or comparable ethical standards. This article does not contain any studies involving animals that were performed by any of the authors. This study was approved by the Ethics Committee of Dr. Lutfi Kirdar Kartal City Hospital, Istanbul (No. 514.179.27).

This retrospective study was performed on patients with subtrochanteric fractures referred to Dr. Lutfi Kirdar Kartal City Hospital, Istanbul.

We retrospectively evaluated 85 patients operated on with diagnoses of subtrochanteric fracture. Patients who visited our clinic for a timely check-up in the postoperative period and had an X-ray in appropriate positions were included in the study. Patients with pathological fractures and vertebral, pelvic, or ipsilateral extremity fractures and patients aged under 18 years or over 65 years were excluded from the study. We excluded a total of 22 patients: four with pathological fractures; seven who were either under 18 or over 65 years; three with pelvic, vertebral, or ipsilateral extremity fractures; and eight with incomplete follow-up, or those who died.

We divided the 85 patients included in the study into two groups. There were 62 patients in Group 1 with IM fixation (IM femoral nail, proximal femoral nail) and 23 patients in Group 2 with EM fixation (95° condylar plate, locked anatomical proximal femoral plate).

We obtained the patients’ historical data from the hospital recording and digital imaging system. Preoperative anteroposterior X-ray of the pelvis; bilateral radiographs of the hip, femur, and knee of the affected side; and CT of the hip and femur were performed on the patients if the fracture was suspected to extend to the piriform fossa. We applied deep vein thrombosis prophylaxis, antibiotic prophylaxis, and skeletal traction to 36 patients (42.3%) based on the amount of displacement in the subtrochanteric fracture. All operations were performed on the traction table, and we mobilized the patients as soon as they were able.

In our study, we noted patients’ age, sex, fractured hip side, etiology, anesthesia type, preoperative waiting time, follow-up period, fracture type, and mechanical complications. In the evaluation of hip function, the results were excellent, good, moderate, or poor according to their Harris hip score (HHS). Because some patients could not be examined, the examination part of the original Harris hip form was removed, and we calculated the modified HHS based on the patients’ self-report, which showed a 99% correlation with the average HHS [6,7]. The obtained score was multiplied by 1.1 and recalculated to be evaluated over 100 points [8]. 

Postoperative radiographs showed varus-valgus angulation and procurvatum (flexion) angulation in the sagittal plane using our hospital’s digital imaging system. Angles below 10° were considered acceptable. In multipart fractures, we evaluated whether there was shortness, if any, by comparing them with the radiographs of the contralateral femur in fractures with displacement or overlap.

We examined the union times of the patients included in the study, observing callus in three of the four cortices on the anterior-posterior and lateral radiographs.

We performed statistical calculations using IBM SPSS Statistics for Windows, Version 22.0 (Released 2013; IBM Corp., Armonk, New York, United States) and used the independent group t-test for statistical analysis. This test indicates whether the difference between the two groups was random or statistically significant. The P-value limit was accepted as 0.05.

## Results

A total of 85 patients were included in the study. The mean age of the patients was 44.13 years and ranged from 16 to 65 years. Overall, 68.2% of the general population were male, and 31.8% were female. There was no difference between the groups in terms of age and gender. The mean patient follow-up period was 13.28 months. Falling from a height was the most common cause of subtrochanteric fracture (Table 1). We applied local anesthesia to 68 patients and general anesthesia to 17 patients.

**Table 1:** Demographic characteristics.

Demographic characteristics	Group 1	Group 2	P-value
Age	46.39	41.87	0.21
Female	20	7	0.95
Male	42	16	0.082
Left side	27	11	0.073
Right side	35	12	0.77
Preoperative time (day)	5.31	4.83	0.68

We classified subtrochanteric fractures according to the Seinsheimer fracture classification. Accordingly, three of the fractures were type 2a, 36 were type 2b, six were type 2c, 15 were type 3a, eight were type 3b, four were type 4, and 13 were type 5 fractures.

We applied the IM femoral nail to 36 patients (42.3%), the PFN to 26 patients (30.5%), the condylar plate with a 95° angle to 13 patients (15.2%), and the proximal femoral anatomical plate to 10 patients (11.7%).

Of the total population, six had varus, one had flexion deformity, five had both varus and flexion, and seven had limb length discrepancy. In Group 1, we detected six varus, one flexion deformity, five varus, and five flexion shortness. In Group 2, we detected no malalignment of more than 10° and only two limb length discrepancy.

We detected late infection in only two patients with a proximal femoral locking plate applied, which we treated by removing the implants. There were seven implant failures, five from Group 1 and two from Group 2. Although we found more delayed union and non-union cases in Group 1, they were not statistically significant. Although malalignment was present in 12 patients (19.3%) in Group 1, we did not detect this in Group 2. We found no significant difference between Groups 1 and 2 in non-union, implant failure, or shortness rates, and no alignment problem was found in the patients who underwent EM fixation. We detected malreduction in 12 of 62 patients who underwent nail (Table 2).

**Table 2:** Comparison of groups in terms of complication rates.

	Group 1, n (%)	Group 2, n (%)	P-value
Delayed union	19 (30.6)	2 (8.6)	0.14
Non-union	3 (4.8)	1 (4.3)	-
Malalignment	12 (19.3)	0	-
Implant failure	5 (8)	2 (8.6)	0.96
Infection	0	2 (8.6)	-
Shortness	5 (8)	2 (8.6)	0.79

We found delayed union in six of 12 patients with malalignment, implant failure in two patients, and non-union in one patient. Of 73 patients without malalignment, 13 had delayed union, three had non-union, and five had implant failure (Table 3). The general population Harris hip score average was 86.5. There was no difference between the groups in terms of HHS.

**Table 3:** Complication rates in patients with and without malalignment.

	With malalignment (12 patients)	Without malalignment (73 patients)	P-value
Delayed union	6	13	0.43
Non-union	1	3	-
Implant failure	2	5	0.64

## Discussion

There is no consensus on which method to use for fixation in subtrochanteric fractures. In our study, we aimed to compare IM and EM methods in these fractures, but we did not detect a significant difference between the two methods. Both methods have advantages and disadvantages. IM methods are associated with less soft tissue damage, support to the medial cortex, and high load-bearing capacity, but the reduction may not be anatomic. In EM methods, anatomical reduction can be achieved but may cause more soft tissue damage and bleeding. Therefore, the surgeon’s experience and preference come to the fore.

Subtrochanteric fractures are not as common injuries as femoral neck fractures or intertrochanteric fractures. This region is under the influence of deforming forces that force the proximal fragment into flexion and abduction. It is also a load-bearing region. Therefore, fractures in this region are injuries that are difficult to treat. There are few studies in the literature comparing treatment options for this type of fracture and which method will be preferred is a controversial issue. We believe that our study will contribute to the literature on this subject.

In our study, we obtained satisfactory clinical results in both groups. Group 1 had a mean HHS of 87.2, while Group 2 had a mean HHS of 84.4. There are similar results to our study in the literature. Mirbolook et al. conducted a retrospective study with 114 patients comparing IM femoral nail and proximal femoral locking compression plates in subtrochanteric fractures and found no significant difference between the two groups’ results [9]. Cook et al. examined 244 subtrochanteric fractures: 168 were fixed with an IM implant and 75 with an EM implant. There was no significant difference between these two groups except for the higher blood transfusion rate in patients with EM implants [10]. Pakuts treated 26 patients, 15 patients with dynamic condylar screw (DCSs) and 11 with gamma nails, and found no significant difference between the two groups in clinical outcomes or complications. However, he stated that patients in the nail-applied group returned more quickly to an active life in the postoperative period [11].

Sowmianarayanan et al. compared the durability of dynamic hip screw (DHS), DCS, and PFN using the finite element analysis method, determining that they all behaved similarly [12]. They compared different IM and IM fixation methods but did not show the clinical superiority of one over the other, similar to our study. Therefore, fixation type remains a controversial issue.

IM nails are strong enough to withstand deforming forces in the subtrochanteric region and the stress from weight in the medial cortex. Umer et al. treated subtrochanteric fractures with PFNs with 94% successful results [13], claiming that PFN could be used in subtrochanteric fractures except for Seinsheimer type 4 fractures. We applied PFN to 26 of 62 patients in Group 1, observing malunion in three patients, implant failure in one, and non-union in two patients. Imerci et al. compared distal femur less-invasive stabilization system (LISS) plates and PFN in their 2015 study involving 32 subtrochanteric fractures. Here, consolidation took longer in the plate group, HHS was higher than in the PFN group, and there was no significant difference in complication and reoperation rates [14].

With the recognition of the biomechanical advantages of IM methods and the development of nail technology, nails are increasingly preferred in treating subtrochanteric fractures. Currently, plates are mostly preferred for complications such as non-union and malunion. In our patient group, non-union developed in four patients, almost all of whom underwent revision surgery with EM fixation with plates.

Proper alignment is an essential issue in the treatment of subtrochanteric fractures. If fixation is performed without proper reduction, some mechanical complications may occur. White et al. conducted a study of 122 subtrochanteric fractures treated with IM nails, showing that rates of non-union and implant failure increased significantly due to implant application with insufficient reduction [15]. In our study, we compared 12 patients with malalignment and 73 patients without malalignment. We found no difference in terms of delayed union, non-union, and implant failure.

Zhou et al. applied IM fixation to a series of 76 patients. Among them, suitable closed reduction was achieved in only five patients who used a traction table, and open reduction with a mini-incision was applied to all the other patients. Proper union was achieved in all patients except for delayed union in one patient [16]. In our study, open reduction and IM fixation were applied to nine patients in Group 1, with one malalignment and two delayed unions.

Riehl et al. treated 35 subtrochanteric fractures with IM nails [17]. Union developed in 97% of the patients without additional surgical intervention. This study examined the type of reduction necessary in IM nailing. More than 10 degrees of angulation were detected in the anterior-posterior or lateral plane in seven of 35 patients. Among these 7 patients, delayed union was found in six patients and non-union in one patient.

Lee et al. compared patients who underwent closed reduction and minimal open reduction in the treatment of subtrochanteric fractures with IM nails. They showed that malalignment and non-union were more common in patients with closed reduction, recommending minimal open reduction if satisfactory results could not be achieved with closed reduction [18].

Malalignment usually takes the form of the proximal part tending to varus, flexion, and external rotation, resulting in the shortening of the abductor’s arm and abductor insufficiency as well as shortening in that extremity, with the more proximal shifting of the trochanter type [19].

DCS is a commonly used implant for subtrochanteric fractures, preferred in oblique or transverse fractures with short proximal fragments. Neogi et al. used this implant in 40 multi-part subtrochanteric fractures and achieved successful results [20]. In our patient group, a 95° dynamic condylar plate was applied to 13 patients, and delayed union was found in one patient with a broken plate and material failure. Rohilla et al. also used DCSs in 43-part subtrochanteric fractures, finding no non-union or implant failure [21].

A proximal femoral locking compression plate is an alternative to the DCS, especially in unstable fractures with short fragments. Previous studies have seen successful results with this implant [22-24]. A proximal femoral locking plate was applied to 10 patients in our study. Deep infection was found in two patients, delayed union in one patient, non-union in one patient, and implant failure in one patient.

As a result, fixation using EM methods in subtrochanteric fractures has disadvantages such as requiring more dissection, more bleeding, prolonged surgery time, and risk of infection. However, it may provide a better reduction. IM methods have advantages such as load-bearing capacity, support for the medial cortex, and being less invasive. However, malalignment is more likely when performed with a closed reduction. The effect of malalignment on the results is also open to interpretation. There is no clear difference between these methods in the literature. We did not detect any significant difference between the two methods in our study. We achieved clinically satisfactory results in Groups 1 and 2 in our series. Future studies might involve larger groups and longer follow-ups, and for now, the surgeon's experience and preference seem to be at the forefront.

The fact that the study is retrospective is a limitation, as it may cause bias in data collection. Although those who did not have an X-ray in a suitable position were excluded from the study, radiological evaluations were made with an X-ray and measurements may be affected by hip position. However, we assume that a similar effect may occur in both groups. Since IM fixation is more preferred, the number of patients in the groups is not close to each other. All patients were operated on in the same clinic but by different surgeons. There is not a single implant in the groups. In the IM fixation group, there are IM femoral nail and PFN. In the EM fixation group, there is a 95° condylar plate and a locked anatomical proximal femoral plate. We think that this is a limitation in terms of homogeneity. A prospective, randomized study with long-term patient follow-up may provide more accurate results in comparing these methods.

## Conclusions

IM and EM fixation methods have advantages and disadvantages in treating subtrochanteric fractures. IM methods are associated with less soft tissue damage, support to the medial cortex, and high load-bearing capacity, but the reduction may not be anatomic. In EM methods, anatomical reduction can be achieved but may cause more soft tissue damage, high infection risk, and bleeding. We obtained similar and clinically satisfactory results in both groups in our study. The issue of fixation methods remains controversial in the literature, and further studies are needed on this subject. IM or EM fixation methods may be preferred in the treatment of subtrochanteric fractures. It is possible to get good results with both methods.
